# The impact of visual cross-modal conflict with semantic and nonsemantic distractors on working memory task: A functional near-infrared spectroscopy study

**DOI:** 10.1097/MD.0000000000030330

**Published:** 2022-09-09

**Authors:** Daisuke Sawamura, Yoshinobu Tanabe, Satoshi Sakuraba, Jiahong Cui, Hiroshi Miura, Ryuji Saito, Masaaki Sugi, Akihiro Watanabe, Yukina Tokikuni, Kazuhiro Sugawara, Mitsunori Miyazaki, Reiko Miyamoto, Shinya Sakai

**Affiliations:** a Department of Rehabilitation Science, Faculty of Health Sciences, Hokkaido University, Sapporo, Japan; b Department of Rehabilitation, Shinsapporo Paulo Hospital, Sapporo, Japan; c Department of Rehabilitation Sciences, Health Sciences University of Hokkaido, Tobetsu, Japan; d Graduate School of Health Sciences, Hokkaido University, Sapporo, Japan; e Department of Rehabilitation, Tokeidai Memorial Hospital, Sapporo, Japan; f Department of Physical therapy, Sapporo Medical University, Sapporo, Japan; g Department of Integrative Physiology, Hiroshima University, Hiroshima, Japan; h Division of Occupational Therapy, Faculty of Health Sciences, Tokyo Metropolitan University, Tokyo, Japan.

**Keywords:** distractor inhibition, functional near-infrared spectroscopy, nonsemantic distractor, semantic distractor, visual cross-modal distractor, working memory

## Abstract

Cross-modal conflicts arise when information from multisensory modalities is incongruent. Most previous studies investigating audiovisual cross-modal conflicts have focused on visual targets with auditory distractors, and only a few studies have focused on auditory targets with visual distractors. Moreover, no study has investigated the differences in the impact of visual cross-modal conflict with semantic and nonsemantic competition and its neural basis. This cross-sectional study aimed to characterize the impact of 2 types of visual cross-modal conflicts with semantic and nonsemantic distractors through a working memory task and associated brain activities. The participants were 33 healthy, right-handed, young male adults. The paced auditory serial addition test was performed under 3 conditions: no-distractor and 2 types of visual distractor conditions (nonsemantic and semantic distractor conditions). Symbols and numbers were used as nonsemantic and semantic distractors, respectively. The oxygenated hemoglobin (Oxy-Hb) concentration in the frontoparietal regions, bilateral ventrolateral prefrontal cortex (VLPFC), dorsolateral prefrontal cortex, and inferior parietal cortex (IPC) were measured during the task under each condition. The results showed significantly lower paced auditory serial addition test performances in both distractor conditions than in the no-distractor condition, but no significant difference between the 2 distractor conditions. For brain activity, a significantly increased Oxy-Hb concentration in the right VLPFC was only observed in the nonsemantic distractor condition (corrected *P* = .015; Cohen *d* = .46). The changes in Oxy-Hb in the bilateral IPC were positively correlated with changes in task performance for both types of visual cross-modal distractor conditions. Visual cross-modal conflict significantly impairs auditory working memory task performance, regardless of the presence of semantic or nonsemantic distractors. The right VLPFC may be a crucial region to inhibit visual nonsemantic information in cross-modal conflict situations, and bilateral IPC may be closely linked with the inhibition of visual cross-modal distractor, regardless of the presence of semantic or nonsemantic distractors.

## 1. Introduction

Information is received via multisensory modalities. We focus or select the necessary information, while we ignore or inhibit unnecessary information for adaptive responses. Cross-modal conflict arises when multisensory information is incongruent. Of the modalities, visual and auditory stimuli are the primary ones.^[1]^

Previous studies investigating audiovisual cross-modal conflicts have focused on visual targets with auditory distractors, and only a few have focused on auditory targets with visual distractors.^[1]^ Studies have investigated the impact of auditory targets with visual distractors using auditory and visual n-back tasks.^[2–4]^ Using stimuli of the same nature across modalities creates semantic competition for maintaining and updating information because numbers have a well-defined semantic organization and are approximately equal in their degree of activating semantic information.^[5,6]^ Semantic distractors generally have a higher interference effect than nonsemantic distractors across categories. However, no study has examined the differences in the impact of visual cross-modal conflict with semantic and nonsemantic competition.

Regarding the mechanism of distractor inhibition, previous neuroimaging studies have reported that the ventrolateral prefrontal cortex (VLPFC), dorsolateral prefrontal cortex (DLPFC), and inferior parietal cortex (IPC) play important roles in both distractor conditions.^[7–9]^ The VLPFC shows hemispheric lateralization that depends on the stimulus modality. The right VLPFC is mainly involved in general inhibition of task-irrelevant distractors and is associated with visuospatial information inhibition,^[7,8]^ while the left VLPFC is mainly linked to semantic and verbal information inhibition.^[7,9]^ Accordingly, we determined these areas as regions of interest (ROIs). The ROI approach is often used to assess the activity of the frontoparietal brain regions, and functional near-infrared spectroscopy (fNIRS) is a neuroimaging technique, in which the ROI approach is used.

fNIRS is a noninvasive device for measuring cerebral cortical hemodynamic activity and can do so in an environment closer to daily life. fNIRS is also much quieter than fMRI and less affected by electrical or magnetic interference from auditory devices. Thus, fNIRS is an ideal imaging technique for auditory research.^[10,11]^

This study aimed to characterize the impacts of semantic and nonsemantic distractors on visual cross-modal conflicts through a working memory (WM) task and assess the correlation between task performance and associated brain activities. Our hypotheses were as follows: first, both distractor conditions would lower task performance, especially in the semantic distractor condition. Second, higher ROI activity would be shown in both distractor conditions than in the no-distractor condition. Additionally, right and left VLPFC activities would be higher in the nonsemantic and in the semantic distractor condition, respectively. Third, brain activity changes in ROIs would correlate with changes in WM task performance. Furthering the basic understanding of brain activity during cross-modal conflict is important for elucidating the neural mechanisms underlying distractibility and the potential to contribute toward future clinical assessment and treatment of distracted patients with brain disorders.

## 2. Methods

### 2.1. Participants

This cross-sectional study, conducted between February 2021 and July 2021, included consecutively recruited right-handed healthy young men from the Health Sciences University of Hokkaido, Tobetsu, Japan. The sample size was calculated a priori using G*power (Universitat Dusseldorf, Dusseldorf, Germany), and the sample size to achieve a.90 statistical power level, based on the results of a previous study,^[12]^ was 27. To be conservative, 15% was added to account for dropouts and outliers, and the determined sample size was 33.

The inclusion criteria were as follows: age range between 20 and 30 years; male sex, considering the sex differences in the WM network^[13]^; and right-handedness. The exclusion criteria were as follows: medical history of neurological and psychiatric disorders; and visual or auditory impairments that affect task performance. Of the 33 participants, 3 were excluded owing to excessive artifacts and device malfunction. Therefore, a total of 30 participants (age range, 20–27 years; mean age,= 22.87 ± 1.94 years; mean score of FLANDERS handedness questionnaire,^[14]^ 9.73 ± .93) were included in the final analysis. The study protocol was approved by the Ethics Committee of the Health Sciences University of Hokkaido (approval number: 20R138128), and written informed consent was obtained from all participants.

### 2.2. Experimental task

**Figure 1.**
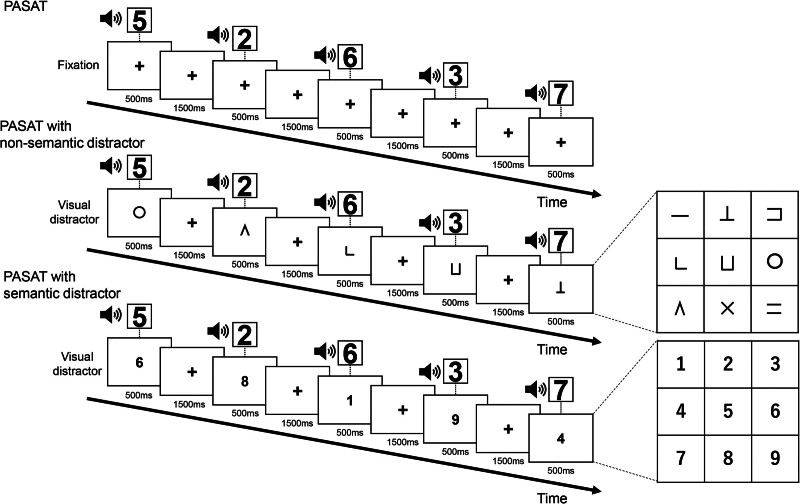
Three types of experiment tasks. The right matrixes indicate all 9 types of distractors. PSAT = Paced Serial Addition Test.

The paced auditory serial addition test (PASAT) is a complex WM test, in which a single-digit number that presents aurally is added to the previously presented number, and is answered orally in a continuous manner. This test was used as a target task. All participants performed the PASAT in 3 conditions: no-distractor, nonsemantic distractor, and semantic distractor conditions (Fig. 1). The no-distractor condition required participants to gaze at the center of the screen and perform the PASAT. In the nonsemantic distractor condition, a set of symbols referring to the WAIS-III Digit Symbol-Coding were used as visual distractors. The auditory target stimuli and visual distractor stimuli were simultaneously presented on the sound speaker and computer monitor using DMDX display software,^[15]^ respectively. In the semantic distractor condition, number stimuli were used as visual distractors. The stimulus sound in the PASAT was set at 70 dB, and fixation or visual distractor stimuli was presented at the center of the screen (stimulus size: 4 cm × 4 cm, visual angle: 3.27°). Each stimulus was presented for 500 ms, followed by a 1500 ms interstimulus interval. The experiment was conducted in a block design, in which a 61.0 seconds PASAT was interleaved with 30.0 seconds rest. The participants completed 9 blocks comprising 3 repeated runs randomly sorted into the 3 task conditions to control for learning effect and fatigue.

### 2.3. Functional near-infrared spectroscopy instrument

**Figure 2.**
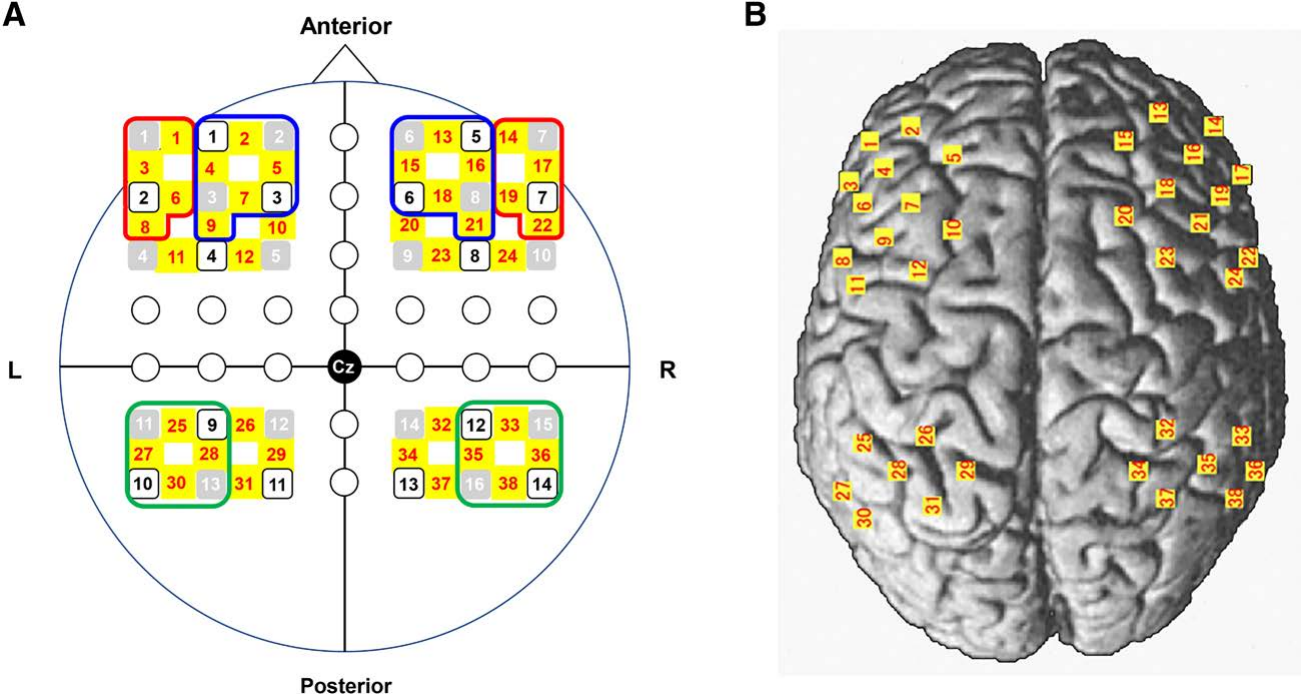
Channel configuration. (A) Gray and white squares show illuminators and detectors; yellow squares show near-infrared spectroscopy channels. Red, blue, and green frames show VLPFC, DLPFC, and IPC, respectively. (B) All channels are placed on the surface of the standard human cortex. DLPFC = dorsolateral prefrontal cortex, IPC = inferior parietal cortex, VLPFC = ventrolateral prefrontal cortex.

A multichannel fNIRS optical topography system (LABNIRS, Shimadzu Corporation Kyoto, Japan) with 3 wavelengths of near-infrared light (780, 805, and 830 nm) was used to measure the hemodynamic changes. The fNIRS probes comprised 16 illuminating and 14 detecting probes arranged alternately with an interprobe distance of 3 cm, resulting in 38 channels according to the international 10 to 20 placement system. The fNIRS optode and reference positions (Cz, Nz, Iz, AL, and AR) were digitized using a 3D digitizer (FASTRAK; Polhemus, Colchester, VT). Figure 2A shows the positions of the probes and channels. The probes were set over the 6 ROIs based on previous studies.^[7–9]^ The 6 ROIs included the bilateral VLPFC (left: channels 1, 3, 6, and 8; right: channels 14, 17, 19, and 22), bilateral DLPFC (left: channels 2, 4, 5, 7, and 9; right: channels 13, 15, 16, 18, and 21), and bilateral IPC (left: channels 25, 27, 28, and 30; right: channels 33, 35, 36, and 38). The anatomical location of each channel was determined according to the Talairach Daemon database^[16]^ (Fig. 2B). The sampling rate was set at 27.8 Hz.

The fNIRS data were analyzed based on the modified Beer–Lambert law to quantify changes in oxygenated hemoglobin (Oxy-Hb). We focused on Oxy-Hb concentration, which is the most representative indication of brain activity.^[17]^ In this study, the baseline period comprised the 6-second period before task onset, and the average Oxy-Hb value of the baseline period was set to zero. The changes in Oxy-Hb concentration during the task were calculated as the difference from the baseline value.^[18]^ In addition, a bandpass filter was applied between 0.01 and 0.3 Hz. Task-related changes in Oxy-Hb concentration in each ROI were averaged over the task period.

### 2.4. Experimental procedure

The experimental procedure consisted of 3 phases. In the practice phase, the participants sat in front of a computer monitor and were instructed to avoid eye, head, and body motion, as well as deep breathing during the NIRS measurements. Subsequently, all participants received instructions for performing the 3 task conditions and practiced each task. During the NIRS recording phase, the Oxy-Hb concentrations were continuously measured. The fNIRS recording phase consisted of 9 blocks for a total of 819.0 seconds. The self-rating phase was the final part of the procedure, where the participants were required to answer their degree of alertness using the Stanford Sleepiness Scale, a self-rating scale that takes approximately 1 minute to complete.

### 2.5. Statistical analysis

A 1-way repeated-measures analysis of variance (ANOVA) or Friedman test was performed to determine the percentage of correct responses in the PASAT and the changes in Oxy-Hb concentration in each ROI according to the normality of the data distribution checked by the Shapiro–Wilk test. The Bonferroni method was used for multiple comparisons. In addition, correlation analyses were performed to examine the relationship between the changes in the percentage of correct responses (Δpercentage of correct responses) and the changes in Oxy-Hb concentration (∆Oxy-Hb) in ROIs in each distractor condition compared to those in the no-distractor condition. We performed Pearson product-moment correlation analysis or Spearman rank-order correlation analysis according to the normality of data distribution. The Δpercentage of correct responses and ∆ Oxy-Hb were calculated by subtracting the value in the no-distractor condition from the value in each distractor condition. Statistical analyses were performed using SPSS (version 25.0; IBM Corp., Armonk, NY), and the α level was set at 0.05.

## 3. Results

### 3.1. Task performance

A 1-way repeated-measures ANOVA revealed a significant difference in task performance among the 3 task conditions (no-distractor: 81.09 ± 12.83%; nonsemantic distractor: 78.49 ± 12.69%; semantic distractor: 76.65 ± 12.87%, F (2, 58) = 11.36, mean squared error (MSE) = 13.126, *P* < .001, *η^2^_p_* = 0.281). A post hoc *t* test showed significantly lower task performances with both nonsemantic and semantic distractors compared with the no-distractor condition (corrected *P* = .029, 95% confidence interval [95% CI]: 0.22–4.99, Cohen *d* = 0.20, and corrected *P* < .001, 95% CI: 2.14–6.74, Cohen *d* = 0.35, respectively). Additionally, no significant difference between the distractor conditions was found (corrected *P* = .199, 95% CI: –0.61 to 4.274, Cohen *d* = 0.14). The mean score of the Stanford Sleepiness Scale was 1.56 ± 0.76 at the conclusion, indicating that the participants were alert during the experiment.

### 3.2. Near-infrared spectroscopy activation

The Shapiro–Wilk test showed that the assumption of the normality was met for Oxy-Hb concentration in each of 3 task conditions in the bilateral VLPFC and IPC (*P* > .05).

**Figure 3.**
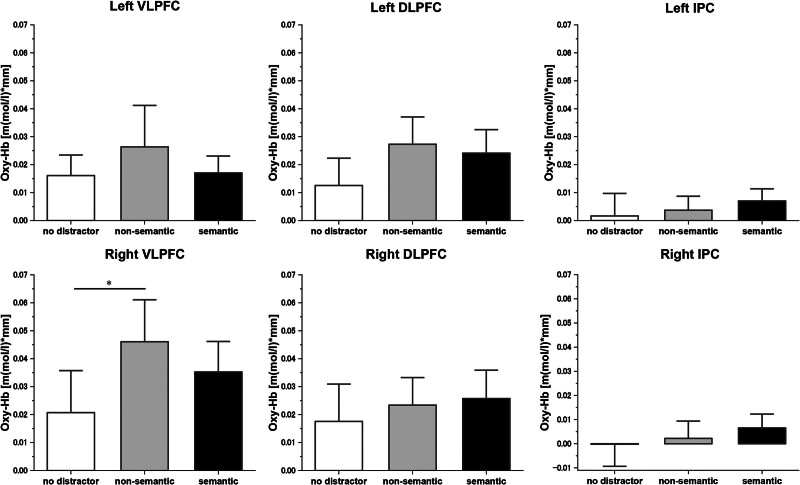
Mean Oxy-Hb concentration during 3 types of experiment tasks. Error bars indicate the standard error. DLPFC = dorsolateral prefrontal cortex, IPC = inferior parietal cortex, Oxy-Hb = oxygenated hemoglobin, VLPFC = ventrolateral prefrontal cortex. *Corrected *P* < .05.

A 1-way repeated-measures ANOVA revealed a significant difference of Oxy-Hb concentration among the 3 task conditions in the right VLPFC (*F* (2, 58) = 5.260, MSE = 0.0009, *P* < .008, *η*^*2*^_*p*_ = 0.154) but no significant differences were found in any other ROIs (left VLPFC: (F (2, 58) = 3.06, MSE = 0.0005, *P* = .055, η2p = 0.095; left IPC: F (2, 58) = 0.62, MSE = 0.0004, *P* = .543, *η*^*2*^_*p*_ = 0.021; right IPC: F (2, 58) = 0.96, MSE = 0.0004, *P* = .390 *η*^*2*^_*p*_ = 0.032; Fig. 3). In the right VLPFC, post hoc *t* test showed significantly increased Oxy-Hb concentration in the nonsemantic distractor condition compared with the no-distractor condition (corrected *P* = .015, 95% CI: 0.04–0.47, Cohen *d* = 0.46), and no significant differences were observed between the semantic distractor and no-distractor conditions (corrected *P* = .181, 95% CI: –0.04 to 0.33, Cohen *d* = 0.35) nor between the semantic distractor and nonsemantic distractor conditions (corrected *P* = .512, 95% CI: –0.30 to 0.09, Cohen *d* = 0.22). In the Oxy-Hb concentration in the bilateral DLPFC that departed significantly from normality in 1 or more of the 3 task conditions, the Friedman test was performed. The latter revealed no significant differences of Oxy-Hb concentration among the 3 task conditions in the bilateral DLPFC (left: χ^2^ (2) = 0.267, *P* = .875, Kendall W = 0.004; right: χ2 (2) = 1.267, *P* = .531, Kendall W = 0.021).

### 3.3. Relationship between the changes in task performance and functional near-infrared spectroscopy activation in the ROIs

**Figure 4.**
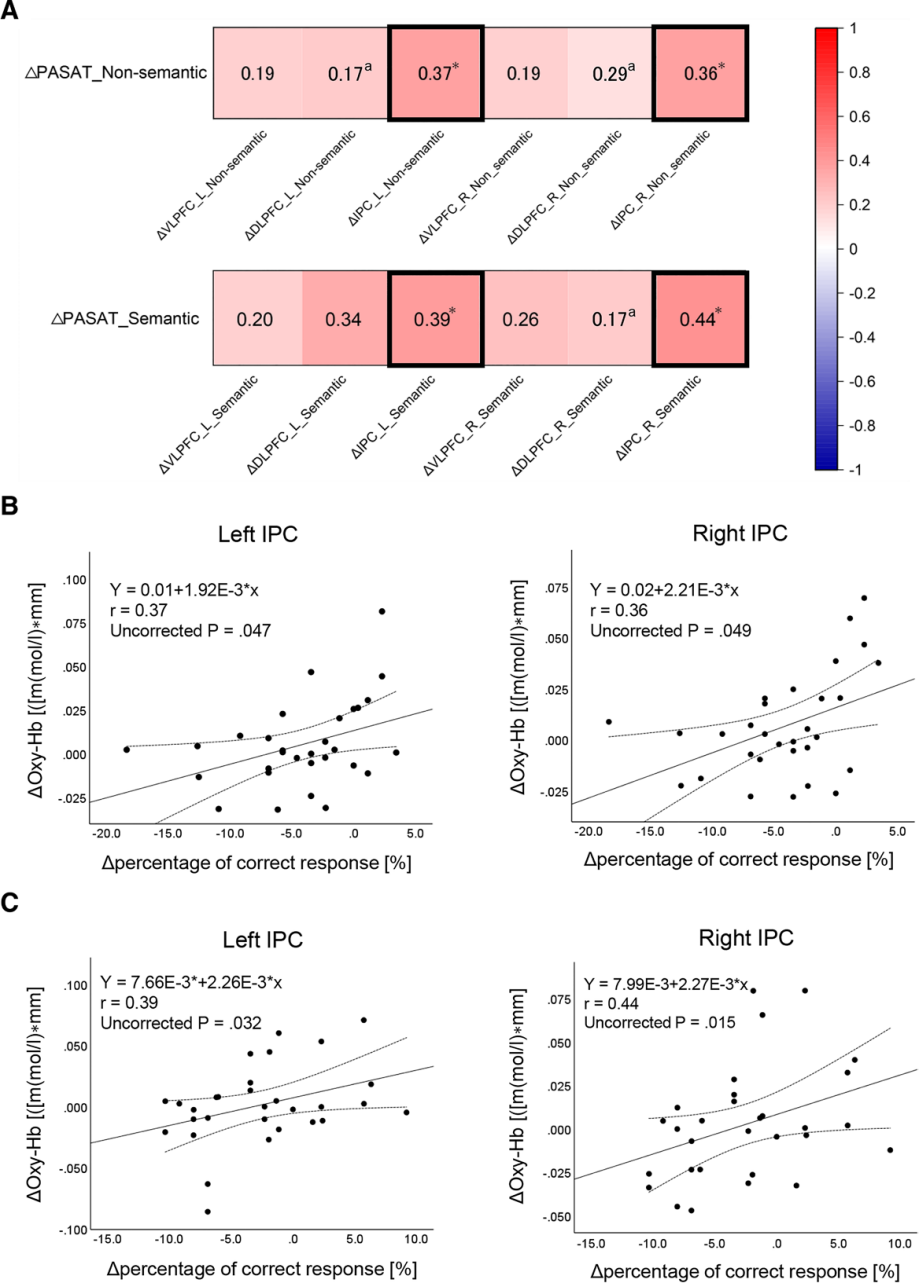
Relationships between the changes in task performance and functional near-infrared spectroscopy activation in each ROI. (A) Overall correlation matrixes. The bold flames show significant correlations. The color bar indicates Pearson correlation coefficient. (B) Scatterplot illustrating the relationship between Δpercentage of correct responses and ΔOxy-Hb in the bilateral IPC in nonsemantic distractor condition. (C) Scatterplot illustrating the relationship between Δpercentage of correct responses and ΔOxy-Hb in the bilateral IPC in semantic distractor condition. DLPFC = dorsolateral prefrontal cortex, IPC = inferior parietal cortex, Oxy-Hb = oxygenated hemoglobin, PASAT = paced auditory serial addition test, ROI = region of interest, VLPFC = ventrolateral prefrontal cortex. *Uncorrected *P* < .05. ^a^Spearman rank-order correlation analysis.

An overview of the correlations between the Δpercentage of correct responses in the PASAT and their ΔOxy-Hb concentrations is presented in Figure 4. Spearman rank-order correlation analyses were only performed for Δpercentage of correct responses and ΔOxy-Hb in the bilateral DLPFC in nonsemantic distractor condition, as well as for Δpercentage of correct responses and right DLPFC in semantic distractor condition because of significant departure from normality. Significant positive correlations were observed between Δpercentage of correct responses and ΔOxy-Hb in the bilateral IPC in both nonsemantic distractor (left: *R* = 0.37, uncorrected *P* = .047; right: *R* = 0.36, uncorrected *P* = .049) and semantic distractor condition (left: *R* = 0.39, uncorrected *P* = .032; right: *R* = 0.44, uncorrected *P* = .015).

## 4. Discussion

The aim of this study was to characterize the 2 types of visual cross-modal conflict with semantic and nonsemantic distractors. To our knowledge, this study is the first to examine the differences between the 2 types of visual cross-modal conflict, focusing on both WM performance and brain activity. In line with our hypothesis, the present study showed that WM task performance was interrupted in both distractor conditions, but no significance was observed between the 2. Regarding brain activity, right VLPFC activity was higher only in the nonsemantic distractor condition, while no significant higher activity was observed in the left VLPFC in the semantic distractor condition. Correlations between changes in task performance and changes in brain activity were found in both distractor conditions only in the bilateral IPC, but not in other regions.

For the PASAT task performance, a significantly lower performance was observed in both distractor conditions than that in the no-distractor condition, as hypothesized. This suggests that visual cross-modal distractors significantly impair auditory task performance regardless of the semantic nature, as previously shown.^[3,12]^ In contrast, no significant differences were observed between the 2 types of conditions, suggesting that visual cross-modal semantic competition does not significantly affect auditory WM performance compared to visual cross-modal nonsemantic competition. This may reflect the characteristics of the number of stimuli selected as target stimuli for this study. Compared to the lexical-semantic competition, the number-derived semantic competition of this study characterized a highly automated way, in which number semantics are accessed, and may result in less effort to actively capture the information.^[6,7]^ In addition, we focused only on task accuracy. Processing speed can be a more sensitive measure to detect the differences between the 2 types of distractors.^[19]^

For brain activity, a significantly increased activation in the right VLPFC was only observed in the nonsemantic distractor condition, in line with our hypothesis. This result suggests that right VLPFC is closely linked with the inhibition of visual object distractor and supports the preference along with right-object laterality trend of VLPFC.^[7]^ Additionally, the lack of increase in left VLPFC activity in the semantic distractor condition may also support the possibility of insufficient or weak semantic competition.

Correlation analyses revealed significant positive correlations between Δpercentage of correct responses and ΔOxy-Hb in bilateral IPC in both distractor conditions. This indicates that increased bilateral IPC activity decreases the interference effect of visual cross-modal conflict, regardless of semantic or nonsemantic distractors. It has been reported that the bilateral IPC lacks hemispheric specialization for inhibiting distractors^[19,20]^ and plays a crucial role in inhibiting visual distractors in neuromodulation^[21]^ and lesional studies.^[22]^

This study has some limitations. First, we investigated only young men. The present study deliberately omitted the effects of sex. The second limitation pertains to the characteristics of the target stimuli. The selection of a target task that causes stronger semantic competition would allow us for more insight into the differences between the 2 types of distractors. The third limitation pertains to the low spatial resolution of the NIRS. Therefore, precise brain areas could not be defined and subcortical areas could not be examined. Future studies should investigate the influence of the population factor, including female individuals and the older population, on the visual cross-modal interfering effect of semantic or nonsemantic distractors after considering the strength of target and distractor stimuli. Furthermore, future research should investigate how each brain region inhibits cross-modal distractors and what functional differences exist between the hemispheres in these brain regions, using neuromodulation techniques.

In conclusion, the visual cross-modal interfering effect of semantic or nonsemantic distractors significantly impaired auditory task performance. Significantly increased activation in the right VLPFC was only observed in the nonsemantic distractor condition, and bilateral IPC was positively correlated with task performance in both types of visual cross-modal conflict. These results suggest that the right VLPFC is a crucial brain area for specialized visual cross-modal conflict with nonsemantic distractors, and bilateral IPC plays a pivotal role in decreasing the interference effect of both types of visual cross-modal distractors.

## Acknowledgments

We hereby acknowledge all participants for their contribution.

## Author contributions

**Conceptualization**: Daisuke Sawamura, Yoshinobu Tanabe

**Data curation**: Daisuke Sawamura, Yoshinobu Tanabe, Satoshi Sakuraba, Jiahong Cui, Hiroshi Miura, Ryuji Saito, Masaaki Sugi, Akihiro Watanabe, Yukina Tokikuni

**Formal analysis**: Daisuke Sawamura, Yoshinobu Tanabe, Satoshi Sakuraba, Jiahong Cui

**Funding acquisition**: Daisuke Sawamura, Shinya Sakai

**Investigation**: Daisuke Sawamura, Yoshinobu Tanabe, Satoshi Sakuraba, Jiahong Cui, Hiroshi Miura, Ryuji Saito, Masaaki Sugi, Akihiro Watanabe, Yukina Tokikuni

**Methodology**: Daisuke Sawamura, Yoshinobu Tanabe, Jiahong Cui, Shinya Sakai, Kazuhiro Sugawara, Mitsunori Miyazaki, Reiko Miyamoto

**Project administration**: Daisuke Sawamura

**Resources**: Daisuke Sawamura, Satoshi Sakuraba

**Software**: Satoshi Sakuraba

**Supervision**: Daisuke Sawamura

**Validation**: Daisuke Sawamura, Satoshi Sakuraba, Kazuhiro Sugawara, Mitsunori Miyazaki, Reiko Miyamoto, Shinya Sakai

**Visualization**: Daisuke Sawamura, Yoshinobu Tanabe

**Writing—original draft**: Daisuke Sawamura

**Writing—review & editing**: Daisuke Sawamura, Shinya Sakai
